# Factors associated with willingness to enter long-term care facilities among older adults in Chengdu, China

**DOI:** 10.1371/journal.pone.0202225

**Published:** 2018-08-16

**Authors:** Ziyue Huang, Qingyue Liu, Hongdao Meng, Danping Liu, Debra Dobbs, Kathryn Hyer, Kyaien O. Conner

**Affiliations:** 1 Department of Health Related Social and Behavioral Science, West China School of Public Health, Sichuan University, Chengdu, China; 2 School of Aging Studies, College of Behavioral and Community Sciences, University of South Florida, Tampa, Florida, United States of America; 3 Department of Mental Health Law & Policy, University of South Florida, Tampa, Florida, United States of America; Medical University Graz, AUSTRIA

## Abstract

**Objective:**

To describe Chinese older adults’ willingness to enter long-term care facilities and to examine individual factors associated with the willingness of using this growing model of long-term care in China.

**Methods:**

A cross-sectional study involving a random sample of 670 adults aged 60+ in the Hezuo community in Chengdu, China in 2016. Respondents were interviewed by trained staff on socio-demographics, health status, quality of life, social support, and willingness to enter long-term care facilities.

**Results:**

Only 11.9% of the respondents were willing to enter long-term care facilities for meeting their medical and social service needs. Multivariable logistic regression analysis showed that willingness to enter long-term care facilities was associated with higher household income (OR = 4.55, 95% CI:1.72–12.00), insurance of Urban Resident Basic Medical Insurance (OR = 4.80, 95% CI:1.17–19.67) and unemployment (OR = 0.48, 95% CI:0.24–0.99). Among those who were willing to enter long-term care facilities, an overwhelming majority (81.2%) would prefer going to a facility within 30-minute walking distance from their current residence, 82.5% indicated the need of nursing care, and 90.0% expected a partnership between the long-term care facility and a large hospital.

**Conclusions:**

A minority of older Chinese were willing to receive long-term care delivered at a facility within walking distance from their current residence. Recent policy aimed at increasing the supply of long-term care facilities may not be consistent with consumer preference for home and community-based care. Balancing investment between home and community-based care, and establishing long-term care insurance remain the top priorities for long-term care research and policy development in China.

## Introduction

China's older adult (60+) population was estimated to be 230 million in 2016 (16.7% of the total population)[1]. One of the characteristics of population aging is the increase in the number of medically vulnerable individuals due to multiple chronic conditions, their treatment, and complications, all of which would increase the demand for post-acute and long-term care.[2–4] It is estimated that more than 20 million older adults would require some forms of long-term care by 2020.

In China, home-based informal care has been the dominant form of long-term care due to filial piety, a traditional cultural value[5]. Family caregiver who sends older adults to long-term care facilities (nursing homes, assisted living communities, etc.) will be regarded as an unfilial person. The issue is further compounded by the fact that nursing homes in China are historically reserved for people with no children, no income, and no known relatives[6]. However, family planning policies implemented in the 1970s to control population growth has resulted in a lack of family caregivers who can provide home-based long-term care. Specifically, the current proportion of the "421/422" families (four grandparents, two parents, and one or two children) caused a major burden on the family informal caregivers[7, 8], with additional challenges coming from the lack of knowledge and training among informal caregivers[9, 10].

In the past few years, there have been more than 144,600 aged care facilities in China and nearly seven million nursing beds for the older adults[11]. Nearly 78.6% of aged care facilities provide medical service in different ways[11]. Doubtlessly, China has made great progress in the development of institutional-based care; but in the face of the acceleration of aging, the supply and demand of long-term care service are still outstanding. The establishment and improvement of facilities with integrated medical and social service have been regarded as one of the important tasks in the health services for the elderly. Therefore, the Chinese government has implemented a series of policies to support the development of institutional-based long-term care as one of the key guidelines put forth by the 13th Five-Year Plan (2016–2020). The State Council published directives on promoting this fledgling industry as part of a concerted effort to meet the growing demand for long-term care[12]. Medical and social service providers are also encouraged to partner with long-term care facilities to provide health and social service[13], with basic principles, development goals, key tasks and safeguard measures of the integration specified by the State Council[14].

In 2005, long-term care facilities that provided both social and medical services appeared in the literature for the first time in China[15]. These facilities could provide health services (medical rehabilitation, etc.) and social services (mental and psychological services, activities, etc.).[16, 17] In recent years, more and more institutional-based long-term care facilities have been established in major cities such as Beijing[18], Shanghai[19], Wuhan[20], Tianjin[21] and Chongqing[22]. The major types of institutional-based long-term care facilities include nursing homes as part of medical centers, medical institutions within senior centers and the newly built institutions offering both medical and social services[23, 24]. These new institutions are similar to the PACE (program of all-inclusive care for the elderly) in the US, integrated care in the UK, and nursing homes in Japan[25–29].

Despite the interest from policymakers, the development of the institutional-based long-term care still faces the following challenges: a weak regulatory framework, the lack of enforcement capacity and funding, and shortage of professional and paraprofessional workers[22, 30]. Therefore, the objectives of this study are to describe older Chinese consumers’ willingness to enter long-term care facilities and to examine individual factors associated with willingness to enter long-term care facilities in China. To our knowledge, this study is among the first to examine willingness to enter long-term care facilities that offer integrated medical and social services among the older people in China.

## Materials and methods

### Ethics consideration

The study was approved by the Sichuan University Institutional Review Board. All participants read a statement that explained the purpose of the survey and written informed consents have been received before being involved in the investigation.

### Participants and sampling

This cross-sectional study was conducted in the Hezuo community, one of six sub-districts within a recently developed urban district in Chengdu[31], Sichuan from January to March in 2016. Chengdu is the capital of Sichuan province and one of the largest metropolises in Western China[32]. All residents aged 60 and above who resided in Hezuo community for at least six months at the time of the survey were eligible to participate in this study. There was a total of 8884 older people (aged 60 and above) in this community as of December 2015. A sample of 710 older people was randomly drawn from the community older adult population using computer “random numbers” generator. During the visits to the respondents’ homes, we first explained the aims of the study to them. After obtaining informed consent, each participant was interviewed by trained staff. The interviews took an average of 20 minutes to complete. Questionnaires were checked after the interviews for completeness. Forty records were excluded from the analysis due to missing information, resulting in 670 final records for the analytic sample.

### Measures

The in-person interview questionnaire contains measures on socio-demographics, willingness to enter long-term care facilities, general wellbeing index, and social support.

#### Socio-demographics

We collected the following information: age, gender, marital status, education, occupation, income (monthly household income per capita), insurance, living condition, being sick in the two weeks before the survey, number and type of chronic diseases, and any hospitalization in the prior year.

#### Willingness to enter long-term care facilities

The interviewer first defined long-term care facilities as institutions that integrate medical and social services in senior care facilities. Second, the interviewer asked whether the respondents are aware of the concept. For those who were not aware, explanations were made to inform them. Respondents were then asked: “Are you willing to enter into one of these facilities to receive integrated medical and social services in the future?” If the answer is “Yes”, additional questions were asked regarding: the most important aspect of choosing a long-term care facility, expectation of travel distance from one’s home to the facility, expectation of monthly costs of the services, expectations of the caregivers, medical staff, services, and quality provided, family support of such care arrangement.

#### General wellbeing index

The WHO-5 items were used to describe the general wellbeing of the respondents about their rating of five statements considering the last 14 days[33]: (1) ‘I have felt cheerful and in good spirits’, (2) ‘I have felt calm and relaxed’, (3) ‘I have felt active and vigorous’, (4) ‘I woke up feeling fresh and rested’ and (5) ‘My daily life has been filled with things that interest me’. The respondents were asked to rate how well each of the five statements applied to him or her. Each of the five items is scored from 0 (none of the time) to 5 (all of the time). The score therefore theoretically ranges from 0 (absence of well-being) to 25 (maximal well-being). A lower total score is associated with depression, poor physical health and psychological health[34].

#### Social support

The Social Support Rating Scale (SSRS)[35] assesses the level of overall social support that each subject received. The SSRS is a self-reported scale including subjective support which reflects the degree of actual support an individual received in the past, objective support which includes perceived interpersonal network that an individual can count on and use of social support which means the degree of individuals using the support. The total score of social support was the sum of all items, with higher score ratings reflecting higher availability of social support. The SSRS total score ranges from 0 to 66 (0–22 = slight social support; 23–44 = moderate social support; 45–66 = extensive social support). The SSRS has been used in a wide range of Chinese populations because of its high reliability and validity, with a Cronbach alpha of 0.92[36].

### Statistical analysis

All survey data were entered into EpiData 3.0. Statistical analyses were carried out using the IBM SPSS version 21.0. We used Pearson’s χ^2^ to examine differences in categorical variables. Multivariate logistic regression was used to examine the relationship between the independent variables. Descriptive analysis was used to describe the specific wishes for the care provided at long-term care facilities among those who were willing to enter one. All p values were two-sided, and a p-value of <0.05 was considered statistically significant.

## Results

Table 1 shows individual characteristics of the sample and differences in these characteristics between positive and negative attitude toward receiving integrated medical and social services in senior care facilities. A total of 670 participants (308 males, 46.0%) were included in this study. The average age of the sample was 69.5 years (SD = 7.6, age ranged 60–99). 76.6% of respondents were married, and 75.1% of the participants reported low education (elementary school or lower). Regarding retirement status, 41.5% were retired and 40.9% were unemployed. 95.3% of the participants were from low (<$375) and middle-income groups ($375-$749). 8.1% and 85.4% of the older people were covered by the Urban Employee Basic Medical Insurance (UEBMI) and the Urban Resident Basic Medical Insurance (URBMI) respectively. 10.9% lived alone while others lived with children or spouse. 15.7% had been sick in the two weeks before the survey, 41.6% of the sample had at least one chronic disease, and 21.2% had been hospitalized in the previous year. 88.5% of the older people had moderate social support and 86.1% of the older people had WHO-5 index scores greater than 13. According to the survey, the proportions of the older people whose preference for the caregivers were self-support, family, community staff and institutional staff were 26.0%, 66.4%, 3.3% and 4.3% respectively.

**Table 1:** Demographic and social characteristics of the older people and factors associated with willing to enter a senior care facility.

Variable	Willing (%)	Unwilling (%)	P value
**Total**	80 (11.9%)	590 (88.1%)	
**Age**			0.240
Age 60–69	46 (57.5%)	333 (56.4%)	
Age 70–79	29 (36.3%)	184 (31.2%)	
≥80 years old	5 (6.2%)	73 (12.4%)	
**Male**	39 (48.8%)	269 (45.6%)	0.595
**Married**	68 (85.0%)	445 (75.4%)	0.058
**Education**			0.013*
No formal education	13 (16.2%)	153 (25.9%)	
Elementary school	40 (50.0%)	297 (50.3%)	
Middle school	12 (15.0%)	91 (15.4%)	
High school or above	15 (18.8%)	49 (8.3%)	
**Occupation**			0.018*
Employed	18 (22.5%)	100 (16.9%)	
Unemployed	21 (26.3%)	253 (42.9%)	
Retired	41 (51.2%)	237 (40.2%)	
**Household monthly income per capita(**$**)**			<0.001*
<$375	48 (60.0%)	457 (77.5%)	
$375-$749	21 (26.2%)	112 (19.0%)	
≥$750	11 (13.8%)	21 (3.6%)	
**Insurance**			0.035*
UEBMI ^a^	65 (81.2%)	507 (85.9%)	
URBMI ^a^	12 (15.0%)	42 (7.1%)	
NCMS ^a^	3 (3.8%)	41 (6.9%)	
**Living condition**			
With children or spouse	77 (96.2%)	520 (88.1%)	0.029*
**Been sick in the prior 2 weeks**	16 (20.0%)	89 (15.1%)	0.256
**Chronic disease**	42 (52.5%)	237 (40.2%)	0.036*
**Hospitalized in prior year**	22 (27.5%)	120 (20.3%)	0.141
**Social support(scores)**			0.081
≤22	3 (3.8%)	16 (2.7%)	
23–44	65 (81.2%)	528 (89.5%)	
≥45	12 (15.0%)	46 (7.8%)	
**WHO-5 index**			0.468
0–12	9 (11.3%)	84 (14.2%)	
13–25	71 (88.8%)	506 (85.8%)	

^a^ UEBMI = Urban Employee Basic Medical Insurance; URBMI = Urban Resident Basic Medical Insurance; NCMS = New Cooperative Medical Scheme;

*p<0.05.

Older Chinese consumers had a poor understanding of long-term care facilities that only 13.6% were aware of such facilities as an option to receive long-term care services. After being explained of the integrated medical and social services that are available in these facilities, only 80 (11.9%) were willing to enter into such facilities should they need the service. The comparisons between people who were willing and those who were not willing showed that they were different in education, occupation, household monthly income, insurance, living condition and chronic disease status (P<0.05).

Table 2 shows the results of the multivariable logistic regression analysis of the willingness to enter long-term care facilities. The final model included three significant variables: unemployment, high household monthly income per capita (≥$750) and insurance of URBMI (P<0.05). The unemployed older people showed more negative attitude towards institutional-based long-term care than those employed people (OR = 0.48, 95%CI:0.24–0.99). The older people with high household monthly income per capita (≥$750) showed more positive attitude compared those older people with low household monthly income per capita (OR = 4.55, 95%CI:1.72–12.00). The older people who had URBMI preferred institutional-based long-term care more than those whose insurances were NCMS (OR = 4.80, 95%CI:1.17–19.67).

**Table 2:** Multivariate logistic regression analysis of willingness to enter senior care facilities among older adults.

Variable	OR ^b^	P-value	95%CI ^b^
**Age(ref = Age 60**–**69)**			
Age 70–79	1.28	0.395	0.73–2.24
≥80 years old	0.76	0.616	0.25–2.26
**Gender(ref = Male)**	1.02	0.945	0.61–1.70
**Marital status(ref = Married)**	0.76	0.520	0.33–1.74
**Education(ref = No formal education)**			
Elementary school	1.13	0.736	0.56–2.27
Middle school	1.07	0.892	0.43–2.65
High school or above	1.56	0.375	0.58–4.20
**Occupation (ref = Employed)**			
Unemployed	0.48	0.049*	0.24–0.99
Retired	0.87	0.684	0.45–1.70
**Household monthly income per capita(**$**)(ref<$375)**			
$375-$749	1.27	0.480	0.66–2.43
≥$750	4.55	0.002*	1.72–12.00
**Insurance(ref = NCMS** ^a^**)**			
UEBMI ^a^	1.94	0.311	0.54–6.97
URBMI ^a^	4.80	0.029*	1.17–19.67
**Living condition(ref = alone)**	3.74	0.091	0.81–17.26
**Been sick in the prior 2 weeks (ref = No)**	1.51	0.226	0.34–1.29
**Chronic disease(ref = No)**	1.56	0.096	0.93–2.63
**Hospitalized in prior year(ref = No)**	1.37	0.310	0.75–2.49
**Social support(scores)(ref≤22)**			
23–44	0.31	0.140	0.07–1.47
≥45	0.58	0.533	0.11–3.20
**WHO-5 index(ref = <13)**	1.37	0.445	0.61–3.10

^a^ UEBMI = Urban Employee Basic Medical Insurance; URBMI = Urban Resident Basic Medical Insurance; NCMS = New Cooperative Medical Scheme for rural residents;

^b^ OR = Odds Ratio; 95% CI = lower and upper 95% confidence interval;

*p<0.05.

Table 3 describes the specific wishes for the care provided at long-term care facilities among those who were willing to enter one. The results indicated that important factors in considering the decision of whether to enter a facility in the descending order of importance: price (46.2%), medical equipment and activities provided (22.5%), services quality (16.2%), facility environment (8.8%), and transportation (5.0%). Regarding convenience, 81.2% of o respondents thought that walking from home to the institutions within 30 minutes is the best. 78.8% of the respondents who were willing to pay for the services less than $300of the average income per month. As for expectation of nurses and doctors, 82.5% of the respondents need professional nursing staff, while 32.5% of the respondents thought that geriatric specialists are necessary, and 53.8% of respondents thought that general practitioners can meet the demand. With respect to expectation of medical services provided, 60.0% of the respondents wanted to obtain medical care services, followed by: regular physical examination services (45.0%), chronic disease management services (18.8%), rehabilitation services (18.8%), health care services (18.8%) and emergency services (16.2%). The proportions of the respondents who wanted the services quality of the institutions to reach primary hospital level, secondary hospital level, and tertiary hospital level account for 23.8%, 51.2% and 22.5% respectively. 48.8% of the respondents said that medical services should be provided in senior centers in the community, 33.8% said social services should be provided in hospitals, and only 17.5% said that medical services should be provided in a long-term care facility. 90.0% of the respondents hoped that the institutions partner with large hospitals.

**Table 3:** Descriptive analysis on the demands for the institutions with integrated medical and social services among the older people.

	Cases	%
**The most important aspect of choosing a long-term care facility**		
Price	37	46.2
Environment	7	8.8
Quality	13	16.2
Medical equipment and activities facilities	18	22.5
Building scale	1	1.2
Traffic	4	5.0
**Expectation of walking distance from one’s home to the facility**		
Not a concern	10	12.5
≤ 30min	65	81.2
31-60min	4	5.0
≥61min	1	1.2
**Expectation of monthly costs of the services (**$**)**		
<$300	63	78.8
$300-$599	16	20.0
$600-$899	1	1.2
≥$900	0	0.0
**Expectations of the caregivers**		
No request	7	8.8
Professional nursing staff	66	82.5
Ordinary domestic workers	5	6.2
Others	2	2.5
**Expectation of medical staff**		
No request	11	13.8
Geriatric specialist	26	32.5
General practitioner	43	53.8
**Expectation of medical services provided**		
Medical care services	48	60.0
Regular physical examination services	36	45.0
Chronic disease management services	15	18.8
Rehabilitation services	15	18.8
Health care services	15	18.8
Emergency services	13	16.2
**Expectation of the services quality of a long-term care facility**		
Tertiary hospital	18	22.5
Secondary hospital	41	51.2
Primary hospital	19	23.8
Others	2	2.5
**Expectation for improvement**		
Services facilities	72	92.3
Quality of staff	58	74.4
Services items	55	71.4
The quality of medical services	58	74.4
**Expectation of the way medical and social services are delivered**		
Hospital-based facilities	14	17.5
Stand-alone facilities in the community	39	48.8
Nursing homes in the community with health services	27	33.8
**Expectation of cooperating with large hospitals**		
Yes	72	90.0
No	8	10.0

## Discussion

The study investigated the Chinese older adults’ attitudes toward receiving integrated medical and social services in senior care facilities in a community in Chengdu. Our findings indicated that only 11.9% of older adults showed positive attitudes towards integrated services. It may due to the strong cultural norms that elderly people thought that only those with no spouse, no living children, or their offspring are not available to provide care should be cared in the institutions[37]. The community, which is a recently urbanized community in Chengdu, is located in the urban-rural district and residents are mostly in “agricultural to non-agricultural” status, so the concept of people in this community is more traditional[31, 38]. And traditionally, most Chinese live in multi-generational families where the elderly are cared by their adult children or extended family[37]. In this study, the vast majority of respondents (92.4%) prefer to be cared at home (including self-support). For the above reasons, the rate of their positive attitude toward institutional-based long-term was low. To improve the attitudes and acceptance among older adults in relation to institutions with integrated medical and social services, the government and social media should vigorously publicize integrated medical and social services[39].

In the current study, the results demonstrated that people with higher household income per capita ($750 and above), employed and covered by Urban Resident Basic Medical Insurance (URBMI) showed more positive attitudes. For older adults, there are three primary resources of income in general: retirement benefits, money from their children and employers[40]. People who are unemployed are more likely to have less income. Besides, only people who have urban registration are eligible to buy URBMI and people who are covered by NCMS are farmers living in rural areas in general, while a study observed a significant income disparity between rural and urban areas[40, 41]. And compared to URBMI, NCMS has lower reimbursement rates. In addition, 46.2% of the people chose the price as the most important factor of the selection of the institutions with integrated medical and social services, and 78.8% of the older people were only willing to spend less than $300 monthly to pay for the services, indicating that the price was an important determinant of choosing facilities-based long-term care. These may suggest that respondents who have higher income and associated socioeconomic status are more willing to purchase services from established providers[42], and people with poor socioeconomic status are at a disadvantage in accessing better health services[43]. But now the government has taken some steps that according to *State Council on the whole Opinions on the Basic Medical Insurance System for Residents in Urban and Rural Areas[41],* URBMI and NCMS will be integrated in the future years, which may help to promote the equity among people in urban and rural areas.

It is worth noting that we did not find associations between willingness to use facilities-based long-term care and health status or general well-being, which were different from those studies showing that older people with a worse health condition or better well-being were more willing to choose institutional long-term care[5, 20, 22]. All significant results in our study were related to economic conditions such as employment status, household monthly income and insurance types. One possible explanation was that most people are not familiar with institution-based long-term care and they seem to associate the formal setting with higher costs (quality). While the government has allocated more funding to the field of long-term care and provides constructions subsidy every occupied bed in some cities, public funding is still limited. There is no national health insurance or long-term care insurance program for older people, such as Medicare in the United States, existing in China[5], and China still does not extend health insurance to long-term care services[40] until now. Therefore, the government should provide nursing homes with the per capita investment and policy support to solve the problem of increasing costs of the older people[9], and build a long-term care insurance system for the older people. Besides, financial support for childless elders and those low-income and low-consuming groups should be increased to enhance their opportunities into the institutions with integrated medical and social service[44].

Results of specific wishes for the care provided at long-term care facilities among those who were willing to enter one have also been illustrated. As for expecting services offered in the facilities, 60.0% wanted medical care services, 45.0% wanted regular physical examination services, 18.8% hoped to access to chronic disease management services, rehabilitation services and health care services, and 16.2% would like to have emergency services. The older people with different health status have different demands for medical services, so the institutions should evaluate their specific needs[24]. Therefore, the government should establish elderly care institutions which meet elders’ needs and if it is possible, take into account personal interests and preferences[30, 45], and formulate standardized health assessment instruments. According to their assessment, the institutions should make suitable health management plans and provide social activities scheme for them. The demand for high quality of consumer-directed services is very high, which is consistent with the literature on older adults in developed countries[46].

According to the survey, results revealed 53.8% of the older people hoped that the general practitioners provide medical services for them, and 82.5% of the older people need professional nursing staff to provide care. However, there was a report that showed the country needs 10 million nursing staff in elderly institutions, but there were only 30,000 qualified persons with national professional certificates[47], and most care workers were poorly paid[48] and inadequately trained[49]. Therefore, the government should encourage the general practitioners and professional nursing staff to work in the institutions to provide integrated medical and social services in terms of the extreme lack of nursing staffs with qualifications for the older people now, and it is necessary to develop a professional long-term care workforce. Besides, the government should increase nurses’ salary in order to attract more professional nurses engaging in geriatric care[30, 48].

In addition, the study indicated that the largest number of respondents wanted to receive home-based care (including self-support, 92.4%), which was consistent with the previous study. For example, a study in the northern city of Tianjin showed 95% of the older people were willing to live at home[50] and Bilsen’s study found older people required care at home[51]. The Aging in Place model where clients delivered the timing and intensity of health and personal care services to them directly in their home in the US[52] may give some directions. In some cities in China, the family (community) doctor contract system that is now practiced community health service center may be a good choice for the delivery of long-term care. The government had made clear that the institutions with integrated medical and social services should be based on community medical services to establish systems of long-term care. Community-based primary care is a key to a sustainable, equitable and efficient healthcare system[53]. Additionally, the community plays a "gatekeeper" role in the older people’s health in integrated care[54] and ensures that residents live in a familiar environment and use the services provided locally to achieve good health and access the services within 30 minutes[55]. The survey shows that 81.2% of the older people want to walk from home to the institutions within 30 minutes, and 48.8% of the people recognize setting up nursing centers in the community. For the above reasons, community- and home-based services appear to be an appealing alternative for older adults who prefer to live in the community and maintain China’s cultural norms[37]. However, in developing community-based services, communities still face multiple barriers. Some communities lack personnel, funds and equipment[56, 57], which can only provide basic medical services for the older people[58] and lack of cohesion within the community may make it difficult for the community to maintain the necessary level of service delivery[59]. Regarding medical care, the items could be added, including the increase of specialist care groups for Parkinson disease, Alzheimer’s disease, and other chronic diseases[30]. According to the survey, 90.0% of respondents wanted the institutions to cooperate with large hospitals. For the above reasons, the government should improve the two-way referral system, and build a "government–CHC- community” elderly care mechanism for integrated medical and social services to better use health services resources of the community[30].

This study has several strengths, including focusing on an “agricultural to non-agricultural” group and taking specific demands for institutions of integrated medical and social service into account. However, several limitations need to be considered. First, the low demand rate for long-term facilities makes the sample size small when analyzing specific demands of the older people. Second, in order to be more persuasive and better consider the development of institutional long-term care, the age groups should include middle-aged people like those in their 50s.

## Conclusions

In conclusion, the proportion of community-living older adults who would enter in long-term care facilities was low. Moreover, the present study suggests that monthly household income, employment status and insurance with URBMI greatly influence the attitudes of the institutions among the older people. Further studies are needed to concentrate on how to set up a suitable long-term care insurance system. The demands of the services from consumers’ aspect provide evidence that should be considered by the government when developing institutional-based long-term care in China. Moreover, home and community-based care should be still considered as main patterns in aged care system.

## Supporting information

S1 Appendix Questionnaires of the study. (PDF)

S1 Dataset (SAV)

S1 Checklist STROBE—Vet statement checklist. (PDF)
