# Supplementary material for: Factors associated with willingness to enter long-term care facilities among older adults in Chengdu, China
Source: PLoS One. 2018 Aug 16;13(8):e0202225. doi: 10.1371/journal.pone.0202225 (PMC6095533; doi:10.1371/journal.pone.0202225)
Supplement: S1 Appendix — (PDF) [file pone.0202225.s001.pdf]

Questionnaires

Q1 Questionnaire of socio-demographics

1. Gender	1) Male 2) Female
2. Age	
3. Your marital status	1) Married 2) Divorce/Widower/Unmarried
4. Your education level	1) No formal education 2) Elementary school<br>3) Middle school<br>4) High school or above
5. Household monthly income per capita (￥)	
6. Now, your employment status	1) Employed 2) Not working 3) Retired
7. What's the type of your insurance?	1) Urban Employee Basic Medical Insurance (UEBMI)<br>2) Urban Resident Basic Medical Insurance (URBMI)<br>3) New Cooperative Medical Scheme (NCMS)
8. Were you sick in the 2 weeks prior to the survey?	1) Yes 2) No
9. Do you have chronic diseases?	1)Yes 2) No
10. Were you hospitalized in prior year?	1)Yes 2) No
11. Whom are you living with？	1) Living alone 2) With children or spouse

Q2 Questionnaire of the WHO-5 items

1=All of the time 2=Most of the time 3= More than half the time
4=Less than half the time 5=Some of the time 6= At no time

	1	2	3	4	5	6
I have felt cheerful and in good spirits						
I have felt calm and relaxed						
I have felt active and vigorous						
I woke up feeling fresh and rested						
My daily life has been filled with things that interest me						

Q3 The Chinese version of the Social Support Rating Scale (SSRS)

1. How many close friends whom you can get support and help from do you have?

1) None 2) 1~2 3) 3~5 4) 6 or above 6

2. Over the past year, you

1) lived alone and were away from families
2) often changed the residence and lived with strangers for most of the time
3) lived with classmates, colleagues or friends
4) lived with families

3. You and your neighbors

1) never care about each other
2) may care slightly when meet with difficulties
3) some neighbors very care about you
4) most of neighbors very care about you

4. You and your classmates/colleagues

1) never care about each other
2) may care slightly when meet with difficulties
3) some classmates/colleagues very care about you
4) most of classmates/colleagues very care about you

5.1 You get support and care from your spouse (lover).

1) None 2) Very little 3) General 4) Fully support

5.2 You get support and care from your parents.

1) None 2) Very little 3) General 4) Fully support

5.3 You get support and care from your children.

1) None 2) Very little 3) General 4) Fully support

5.4 You get support and care from your siblings.

1) None 2) Very little 3) General 4) Fully support

5.5 You get support and care from other family members (such as sisters-in-law).

1) None 2) Very little 3) General 4) Fully support

6. In the past, when you were in a difficult situation, the source of the economic support and practical help was from

1) None
2) The following (can select multiple items):
A. Spouse B. Other family members C. Friends D. Relatives
E. Classmates/Colleagues F. Company/School G. Official or semi-official organizations
H. Unofficial organizations I. Others_____

7. In the past, when you were in a difficult situation, the source of comfort and concern was from

1) None
2) The following (can select multiple items):
A. Spouse B. Other family members C. Friends D. Relatives
E. Classmates/Colleagues F. Company/School G. Official or semi-official organizations
H. Unofficial organizations I. Others_____

8. When you are in trouble, you talk to

1) Nobody 2)1~2 people who is/are very close to you

3) friends if they ask you	4) people initiatively
9. When you are in trouble, you ask for help	
1) No 2) Seldom 3) Sometimes 4) Always	
10. You participate in group activities.	
1) Never 2) Occasionally 3) Frequently 4) Initiatively and actively	

Q4 Questionnaire of willingness to enter long-term care facilities

1. What's your expectation of care services?
1) Self-support 2) Home-based care 3) Community-based care 4) Institutional care
2. Do you know long-term care facilities?
1) Do not understand 2) Basically understand 3) Fully understand
3. Do you have the willingness to enter long-term care facilities?
1) Yes 2) No
4. For you, the most important aspect of choosing a long-term care facility:
1)Price 2) Environment 3) Quality 4) Medical equipment and Activities facilities<br>5) Building scale 6) Traffic
5. Expectation of travel distance from one's home to the facility:
1) No a concern 2) ≤ 30min 3) 31~60min 4) ≥61min
6. Expectation of monthly costs of the services（￥）:
1) <￥2000 2) ￥2000~￥3999 3) ￥4000 ~￥5999 4) ≥￥6000
7. Expectations of the caregivers:
1) No request 2) Professional nursing staff 3) Ordinary domestic workers 4) Others_______
8. Expectation of medical staff:
1) No request 2) Geriatric specialist 3) General practitioner
9. Expectation of medical services provided:
1)Medical care services 2) Regular physical examination services<br>3) Chronic disease management services 4) Rehabilitation services<br>5) Health care services 6) Emergency services
10. Expectation of the services quality of a long-term care facility:
1) Tertiary hospital 2) Secondary hospital 3) Primary hospital 4) Others____
11. Expectation for improvement:
1) Service facilities 2) Quality of staff 3) Service items 4) The quality of medical services
12. Expectation of the way medical and social services are delivered:
1) Hospital-based facilities 2) Stand-alone facilities in the community<br>3) Nursing homes in the community with health services
13. Do you expect long-term care facilities cooperating with large hospitals?
1) Yes 2) No
